# Influence of the Fibrous Network Architecture on the Mechanical Properties of Melt-Blown Non-Woven Thermoplastic Polyurethane Fabrics

**DOI:** 10.3390/polym18070857

**Published:** 2026-03-31

**Authors:** Qunsong Wang, Ming Lu, Rimin Zhou, Mingkun Li, Chao Ding, Yun Liang

**Affiliations:** 1 National Engineering Research Center of Papermaking and Pollution Control, South China University of Technology, Guangzhou 510641, China; wangqunsong2@kingfa.com.cn; 2 Kingfa Science & Technology Co., Ltd., Guangzhou 510663, China; luming@kingfa.com.cn (M.L.); zhourimin@kingfa.com.cn (R.Z.); limingkun@kingfa.com.cn (M.L.)

**Keywords:** thermoplastic polyurethane, melt-blown, non-woven fabrics, multiple linear regression, finite element modeling

## Abstract

Melt-blown thermoplastic polyurethane (TPU) non-woven fabrics are increasingly valued in biomedical and industrial applications due to their elasticity, breathability, and skin compatibility. However, the relationship between their microscale fibrous network architecture and macroscopic properties remains insufficiently understood. This study investigates the influence of fiber diameter, porosity, and grammage on the mechanical properties of TPU non-woven fabrics through experimental characterization and finite element modeling (FEM). TPU pellets were melt-blown into fabrics with controlled structural variations, and their properties were analyzed using scanning electron microscopy (SEM), tensile testing, and air permeability measurements. Multiple linear regression (MLR) revealed that increased grammage predominantly enhances tensile strength (transverse *β* = 0.764, longitudinal *β* = 0.899), while fiber diameter and porosity affect transverse and longitudinal elongation in a different way. FEM simulations based on three-dimensional fiber networks further validated these relationships, demonstrating that increased porosity (e.g., from 85% to 92.5%) reduces tensile stress by over 50%, whereas larger fiber diameters (e.g., from 2 μm to 14 μm) decrease elongation at break by approximately 70%. By integrating experimental and computational approaches, this research provides valuable insights for optimizing TPU non-woven fabrics to meet specific performance requirements in wound dressings and other advanced applications.

## 1. Introduction

Non-woven wound dressings are categorized into medical bandages, wound contact layers, and adhesive plasters, among others. The non-woven textile backing, serving as the primary substrate, critically determines the overall physical performance of these dressings, including flexibility, moisture management, and mechanical durability. A wide range of materials is used for this purpose, including synthetic polymers [1,2] such as polyethylene (PE), polypropylene (PP), and polyester (PET), which are widely used in commercial wound dressings. In addition, biodegradable synthetic polymers [3,4,5] (like polylactic acid, poly-ε-caprolactone and polyglycolic acid) and natural biopolymers [6,7] (such as chitosan and alginate) are also gaining more and more interest in this field due to their biocompatibility, biodegradation and ability to promote wound healing. Among these, TPU non-woven fabrics have emerged as the preferred choice for advanced wound care due to their intrinsic elasticity, skin-friendliness, and breathability [8,9,10], which is essential for conforming to dynamic human contours while maintaining microbial barrier functions. In addition, TPU-based non-wovens have garnered significant attention in advanced filtration, biomedical materials, and flexible composites due to their unique elasticity, tunable porosity, and three-dimensional fibrous architecture [11,12,13].

Non-woven fabrication techniques are broadly classified based on the state of the polymer during fiber formation [14]. The primary categories include melt-spinning processes and solution-based spinning processes. In melt-spinning, such as melt-blowing [15,16] and spunbonding [17,18], molten polymer is extruded and attenuated. In solution-based spinning processes, such as electrospinning [19,20] and solution-blow spinning [21,22], a polymer solution is used to generate fibers. Among these, the melt-blown method, a subset of melt-spinning, has become an industrially dominant approach owing to its high production efficiency, minimal solvent waste, and cost-effectiveness for mass production. For instance, polypropylene (PP) is the predominant material for commercial melt-blown non-wovens, primarily due to its straightforward rheology (low melt viscosity) and well-established, stable processing protocols [23,24,25,26]. In contrast, the melt-blowing of polymers with complex chemical structures like TPU—characterized by alternating hard/soft segments and temperature-sensitive rheology—is much more sophisticated and less standardized. The process is prone to unstable fiber formation, making it challenging to consistently achieve the uniform fiber morphology and targeted three-dimensional network structures that are readily attainable with PP. While conventional studies primarily focus on optimizing melt-blown process parameters (e.g., air pressure and die temperature) to regulate fiber morphology [25,27,28,29], systematic investigations focusing on the relationship between microstructural characteristics, like fiber diameter, and macroscopic properties, like tensile behavior, remain underexplored. This knowledge gap critically limits the rational design of high-performance TPU non-wovens for practical applications.

To address this, our study systematically investigates how fiber diameter, porosity, and network geometry influence the mechanical properties of TPU non-woven fabrics. For this purpose, raw TPU material was characterized and then processed into non-wovens via melt-blowing. Fibrous network architecture and physical properties like grammage, tensile behavior, and air permeability were analyzed systematically. Based on experimental results, structure–property relationships were established by multiple linear regression. Finally, finite element modeling (FEM) of a 3D fiber network was used to verify the structure–property relationships while eliminating interference from other non-structural factors.

Unlike conventional research, which simply investigates how processing parameters influence the properties of non-woven fabrics, this study involves an integrated experimental–statistical–computational methodology to understand the inherent link between the fiber architecture and macroscopic properties of non-wovens. Specifically, the FEM model with a 3D fiber network in this study is a significant advancement over commonly used 2D models, as it explicitly accounts for critical three-dimensional phenomena, such as fiber entanglement, buckling, and through-thickness stress distribution. This study aims to provide practical guidelines for optimizing the structure design of TPU non-wovens in biomedical as well as industrial applications.

## 2. Methods

### 2.1. Melt-Blown TPU Non-Woven Fabrics

The study utilized a commercial, elastomeric thermoplastic polyurethane (TPU) supplied by Kingfa Sci. & Tech. Co., Ltd. (Guangzhou, China). A full characterization of its chemical, thermal, and mechanical properties is provided in Section 3.1. The TPU pellets were first dried at 90 °C for 4 h to remove residual moisture before processing. The melt-blowing process was conducted using a specific melt-blowing apparatus equipped with a single-screw extruder (Nanjing Keerte Mechanical Equipment Co., Ltd., Nanjing, China). The parameters of the melt-blown extruder are as follows: the length-to-diameter ratio (L/D) was 30:1, the screw diameter was 90 mm, and the compression ratio was 3:1. The TPU pellets were fed into the extruder, where they were melted at a controlled temperature ranging from 200 °C to 230 °C. The molten polymer was then extruded through a linear die consisting of about 3000 orifices with a diameter of 0.3 mm, spaced at 0.65 mm intervals. The die pressure was 3 MPa, and the die temperature was 240–260 °C. High-velocity hot air with a temperature of 250 °C and pressure of 30–40 kPa (corresponding to a frequency setting of 30–40 Hz) from an air knife was immediately blown onto the extruded polymer streams to attenuate them into microfibers. The resulting non-woven web was collected on a rotating drum collector placed at a certain distance (10–20 cm) from the die, with an adjustable collector speed of about 3–8 m/s to control fiber alignment and web density. The process parameters, including air pressure, die temperature, and collector distance, were adjusted to achieve uniform fiber morphology.

### 2.2. Test Methods

The degree of microphase separation in TPU was analyzed using a Fourier transform infrared spectrometer (FTIR; Nicolet IS50, Thermo Fisher Scientific, Waltham, MA, USA). FTIR spectra of raw TPU material were obtained in the range of 4000 cm^−1^ to 400 cm^−1^ using the attenuated total reflectance (ATR) mode. The spectra were subsequently analyzed with Omnic 8.2 software.

To investigate the thermal properties of TPU raw materials, a differential scanning calorimeter (DSC; DSC 214, NETZSCH, Selb, Bavaria, Germany) was employed. Approximately 7 mg of the TPU raw material was used. The polymer was initially held at 30 °C for 3 min, then heated to 250 °C and maintained for 5 min. It was subsequently cooled to 30 °C, held for 3 min, and heated again to 250 °C. Both the heating and cooling rates were set at 10 K/min, and all measurements were conducted under a nitrogen atmosphere.

Thermogravimetric analysis (TGA, TG 209 F1, NETZSCH, Selb, Bavaria, Germany) was performed to examine the thermal stability of TPU raw materials. Samples weighing about 20 mg were heated from room temperature to 750 °C at a rate of 15 °C/min. Nitrogen gas was used during the test at a flow rate of 50 mL/min.

Porosity of non-woven fabrics, *φ*_v_, was calculated using the following equation:

(1) $$\varphi_{v}=1-\frac{\rho_{s}}{\rho_{0}}$$

Here, $\rho_{s}$ is the apparent density calculated from the ratio between the weight and volume of TPU non-woven fabrics; $\rho_{0}$ is the density of TPU bulk material, which was determined to be 1.21 g/cm^3^ by a solid densitometer (Shenzhen Dahometer Density Measurement Instrument Co., Ltd., Shenzhen, China). Six pieces of non-woven fabrics (3 cm × 3 cm) were stacked to reduce errors, and the thickness of the non-woven fabrics was measured using a film thickness gauge.

The morphology of TPU non-woven fabrics was examined using a scanning electron microscope (S-3400N, Hitachi, Tokyo, Japan). The working distance of the SEM was about 8 mm, and the working voltage was 10 kV with automatically corrected contrast and brightness. The magnification was 2000. Prior to scanning, the samples were gold-coated via sputtering to enhance conductivity (10 mA, 120 s). The fiber diameters and pore sizes were subsequently quantified using Image J software (Version 1.8.0).

The mechanical properties of TPU non-woven fabrics were evaluated with an electronic universal testing machine (Instron 5966, Norwood, MA, USA). For the tests, the fabrics were cut into rectangular specimens measuring 15 cm × 5 cm with a thickness of about 0.4 mm. Six specimens from each group were tested. The tensile testing speed was set at 15 mm/min.

The grammage of the non-woven fabrics was determined according to ISO 9073-1 [30]. Five square specimens (100 cm × 100 cm) were cut, and their mass was measured using an analytical balance (Mettler Toledo MS104TS, Greifensee, Switzerland). The grammage (in g/m^2^) was then calculated.

Air permeability was evaluated following ISO 9237 [31] using a Textest FX3300 IV (Schwerzenbach, Switzerland) permeability tester. Circular specimens (20 cm^2^ test area) were subjected to a 100 Pa pressure differential under standard conditioning. Five measurements per sample were taken at different locations.

### 2.3. Finite Element Model

Finite element modeling was used to analyze the influence of fibrous network architecture on the mechanical properties of TPU non-woven fabrics. First, a 3D fiber network was generated by Python (Version 3.10.2) code. The core idea of generating a 3D fiber network involves randomly selecting start and end points for fibers on the two random surfaces of a defined 3D space after verifying that the distance between new and existing fibers meets the required criteria. Then, the geometric models of the fibers were created by defining their diameters. This process was iterated until the desired porosity was achieved. Material properties, including elastic modulus and Poisson’s ratio, were also defined in the code according to the experiment results. For a fiber with a diameter of 6 μm, approximately 200 fibers were generated in a cube with dimensions of 300 μm × 300 μm × 150 μm.

Finite element analysis was performed in ABAQUS (Version 2023, Dassault Systèmes, Inc., Vélizy-Villacoublay, France) software. A single fiber was meshed using hexahedral C3D20 elements for the finite element simulations. With one end of the non-woven fabric fiber network fixed, a certain displacement was applied to the other end, and a maximum stress value of the mesh was defined as the fracture criterion. The material of fibers was assumed to be isotropic and elastoplastic. Mechanical properties were defined according to experimental results. To analyze the effect of fiber diameters on mechanical properties, fiber diameters were chosen to be 2, 6, 10 and 14 μm, respectively, with 90% porosity. To analyze the effect of porosity on mechanical properties, porosities were chosen to be 85, 87.5, 90, and 92.5%, respectively, with a fiber diameter of 6 μm.

## 3. Results

### 3.1. Physical Properties of TPU Raw Materials

FTIR was used to analyze the degree of microphase separation. The absorption band at 3332 cm^−1^ is attributed to the stretching vibration of N-H [32]. The absorption bands at 1218 cm^−1^ and 1072 cm^−1^ are attributed to the asymmetric and symmetric stretching vibrations of C-O groups in the ester linkages, confirming that the TPU used in this study is polyester-based [33]. The absorption band at 1597 cm^−1^ is attributed to the C=C skeletal vibration of the aromatic ring, confirming that the TPU used in this study is aromatic-type [34].

The characteristic absorption region of C=O groups in the 1660–1780 cm^−1^ range represents a crucial spectral window for investigating hydrogen bonding interactions in polyurethane systems. In FTIR spectroscopy, the vibrational frequency of a chemical bond is determined by the force constant of the bond and the reduced mass of the atoms involved, as described by Hooke’s law:

(2) $$v=\frac{1}{2\pi }\sqrt{\frac{K}{\mu^{\prime }}}$$

where v is the wavenumber, K is the force constant, and $\mu^{\prime }$ is the reduced mass, which is calculated by the following equation:

(3) $$\mu^{\prime }=\frac{m_{1}m_{2}}{m_{1}\;+\;m_{2}}$$

When the carbonyl group (C=O) participates in hydrogen bonding (C=O···H–N), the electron density on the oxygen atom is partially shared with the hydrogen atom. This weakens the C=O bond, effectively reducing its force constant K. According to the equation above, a lower force constant results in a lower vibrational frequency (lower wavenumber). Conversely, free (non-hydrogen-bonded) carbonyl groups retain their full bond strength and thus vibrate at a higher frequency (higher wavenumber).

**Figure 1:**
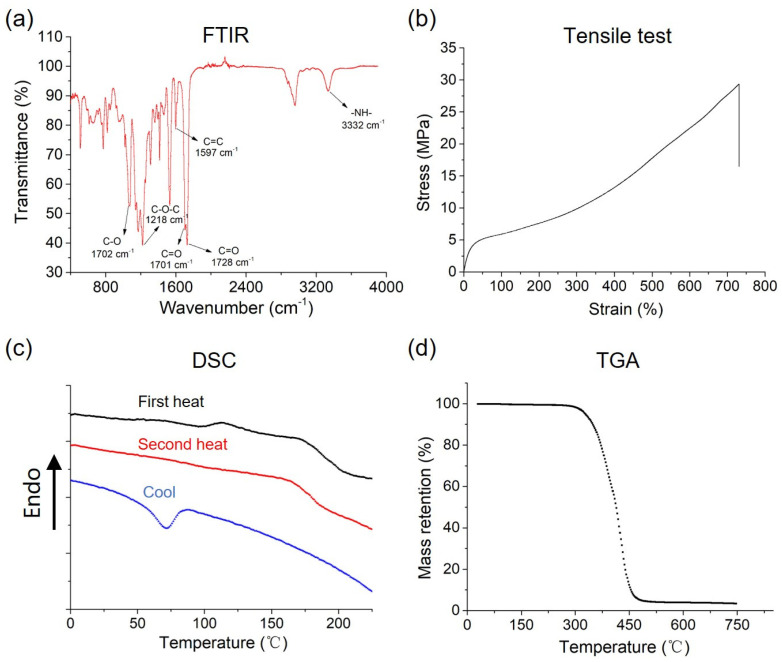
Physical characteristics of TPU raw material. (**a**) FTIR spectrum of the original TPU raw material pellets. (**b**) Tensile test. (**c**) DSC scanning. (**d**) TGA.

As shown in Figure 1a, the carbonyl stretching vibration splits into two distinct bands: the lower frequency absorption at 1701 cm^−1^ corresponds to hydrogen-bonded carbonyl groups (C=O···H-N), while the higher frequency peak at 1728 cm^−1^ arises from free (non-hydrogen-bonded) carbonyl groups. This frequency shift of approximately 30 cm^−1^ originates from the reduced force constant of the C=O bond when participating in hydrogen bonding, consistent with the relationship between hydrogen bond strength and spectral shifts [35].

In typical segmented polyurethanes, the spatial distribution of these carbonyl species reflects the microphase-separated morphology. Urethane groups forming intramolecular hydrogen bonds (NH···O=C) predominantly reside within the hard segment domains, whereas free carbonyl groups tend to localize at the soft–hard phase interfaces or within the soft matrix. This distribution pattern has been confirmed by combined FTIR and AFM studies [36]. The hard segment content (*X_HS_*) can be calculated using the widely accepted equation [37]:

*X_HS_* = *A_HB_*/(*A_HB_* + *A_Free_*) × 100% (4)

where *A_HB_* and *A_Free_* represent the integrated areas of hydrogen-bonded and free carbonyl peaks, respectively. For accurate quantification, spectral deconvolution was performed using appropriate Gaussian–Lorentzian fitting functions after careful baseline correction. The degree of microphase separation of this sample was calculated to be 35%. Notably, the degree of microphase separation serves as a critical parameter correlating with material properties. Previous studies have demonstrated that a higher degree of microphase separation corresponds to improved mechanical properties in elastomeric polyurethanes [38]. These structure–property relationships underscore the importance of precise FTIR analysis in choosing material for melt-blowing.

The typical tensile curve of TPU material is shown in Figure 1b. The TPU material used for melt-blowing shows satisfying mechanical properties, with a tensile stress of 26.7 MPa, an elastic modulus of 17 MPa, and an elongation at break of 734%.

The thermal behavior of the TPU pellets was systematically analyzed via DSC and TGA. DSC measurements (Figure 1c) revealed a melting temperature *(T*_m_) at 159.4 °C. A crystallization temperature (*T*_c_) peak at 71.6 °C was observed during cooling. In Figure 1d, the degradation onset temperature (*T*_d_, defined as 5% mass loss) was observed at 326 °C under dynamic heating, establishing a safety margin above the maximum processing temperature of about 220 °C. These results collectively validate the TPU’s suitability for melt-blowing, as its thermal properties ensure stable fiber formation without decomposition during extrusion or high-velocity air attenuation.

### 3.2. Melt-Blown Process of TPU Non-Woven Fabrics

**Figure 2:**
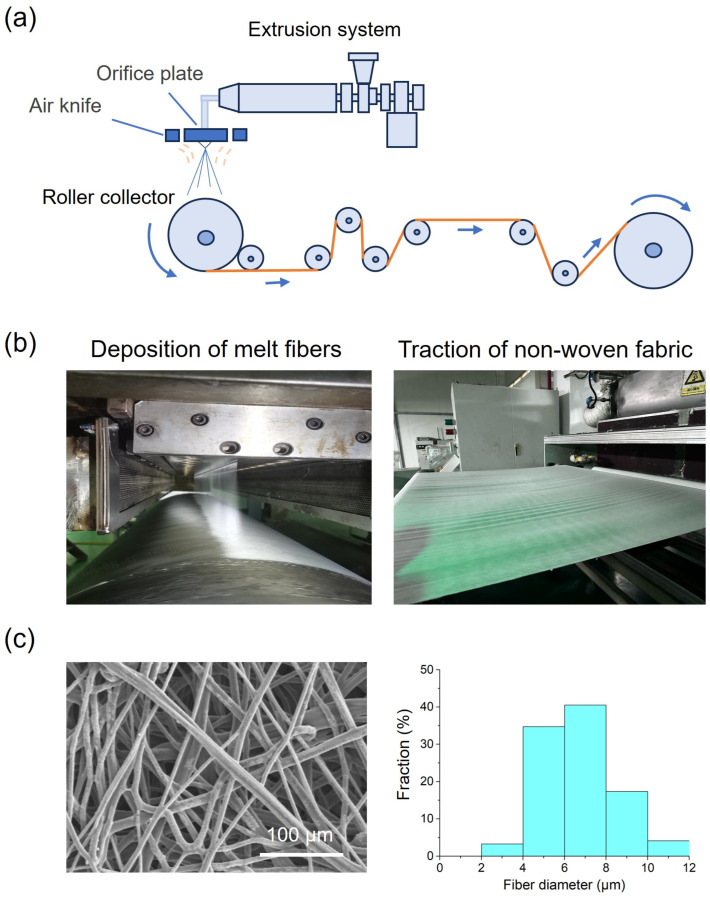
Melt-blown process of TPU non-woven fabrics. (**a**) Schematic of the process of melt-blowing. Orange line indicates the obtained non-wovens. Arrows indicate the moving direction of collector or non-wovens. (**b**) Photos of the spinneret and collector showing the process of deposition of melt fibers (**left**) and traction of non-woven fabric (**right**). (**c**) Typical SEM images of melt-blown fibers (**left**) and the statistical result (**right**) of fiber diameter after analyzing five SEM images with at least 100 fibers.

As shown in Figure 2a, the melt-blown process of TPU involves the extrusion of molten polymer through fine nozzles, followed by high-velocity hot air attenuation to form microfibers. First, dried TPU pellets are fed into a single-screw extruder heated to a temperature range of 180–220 °C, ensuring complete melting while avoiding thermal degradation. The molten polymer is then forced through a linear die assembly containing 3000 orifices with a diameter of 0.3 mm.

Simultaneously, high-temperature compressed air is injected through an air knife, rapidly stretching the extruded polymer streams into ultrafine fibers. This aerodynamic drawing process induces molecular orientation, enhancing fiber strength. The attenuated fibers are subsequently deposited onto a moving drum collector, where they entangle through both mechanical interlocking and residual adhesive properties of the semi-molten TPU.

### 3.3. Fibrous Network Architecture and Physical Properties of TPU Non-Woven Fabrics

Typical SEM images with high and low magnification of fibers in non-woven fabrics are shown in Figure 2c and Figure S1a, respectively. The fiber diameter ranges from 2 μm to 12 μm, with an average size of 6.7 μm. Statistical results in Figure S1b show there was no obvious fiber orientation due to the relatively low rolling speed. By changing melt-blown parameters, fabrics with different diameters ranging from 7.4 μm to 9.5 μm and porosities ranging from 87.2% to 90.1% were prepared, while grammage was kept almost the same. The properties of non-woven samples are presented in Table 1. FTIR of these samples in Figure S2 shows no obvious difference compared with raw materials, indicating the processing stability of TPU materials.

### 3.4. Analysis of the Influence of Fibrous Network Architecture on Mechanical Properties of TPU Non-Woven Fabrics

To quantitatively assess the relationships between key fiber structural parameters (grammage, porosity, and fiber diameter) and mechanical properties (tensile force and elongation at break) of melt-blown non-woven fabrics, multiple linear regression analysis was employed. MLR is a widely used statistical method for modeling the linear relationship between multiple independent variables and a dependent variable. A linear model was chosen over alternative forms (e.g., exponential) based on its simplicity, interpretability, and the absence of prior evidence suggesting strong nonlinear interactions among the studied structural parameters in the examined range. Given the limited sample size (*n* = 5), conventional significance testing (*p*-values) proved unreliable due to insufficient statistical power. Consequently, this analysis focused on interpreting the standardized regression coefficients (*β*) to describe the relative influence of tested factors. Standardized coefficients (*β*) indicate the expected change in the dependent variable (in units of its standard deviation) for a one standard deviation increase in an independent variable, while holding all other variables constant. Removing the influence of measurement units allows comparison of effect sizes across predictors with different scales.

Regression analysis revealed key directional differences in tensile behavior (Table 2 and Table 3). Grammage showed the strongest positive effect on tensile force in both directions (transverse *β* = 0.764, longitudinal *β* = 0.899). For elongation at break, both gram and porosity dominated transverse behavior, while fiber diameter influenced longitudinal behavior most (*β* = 0.600). Notably, grammage negatively affected longitudinal elongation (*β* = −0.152), demonstrating opposite effects on longitudinal behavior. However, the consistently large standard errors suggest these relationships require further verification with larger datasets or other theoretical analysis methods.

### 3.5. Finite Element Modeling of TPU Fabrics

**Figure 3:**
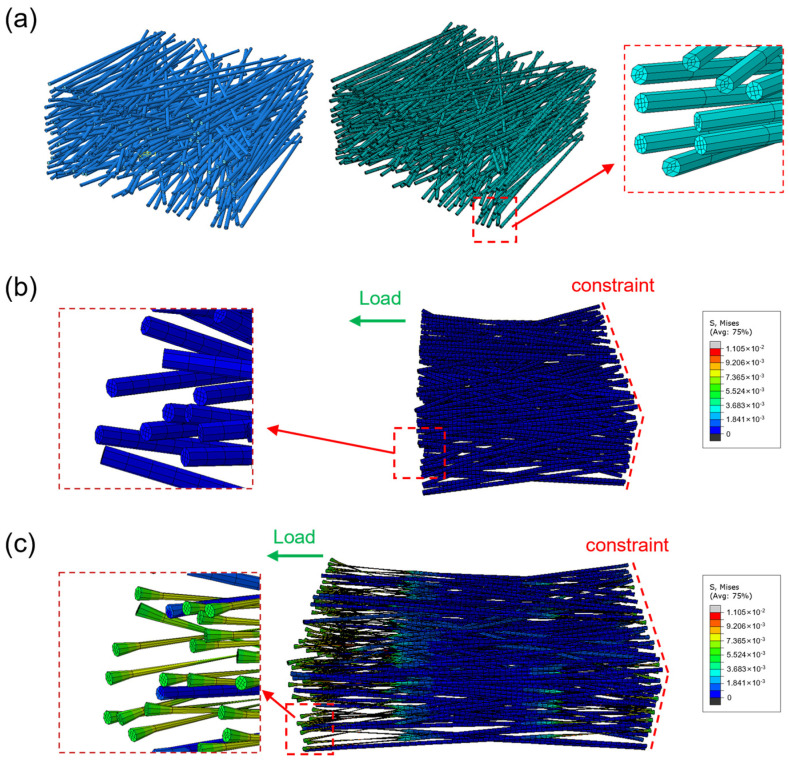
Finite element modeling of TPU fabrics. (**a**) Generation of a 3D model of a fiber network using Python code. Four seed points were distributed along the circumferential direction to generate a swept mesh, resulting in approximately 700,000 hexahedral elements in the final model. (**b**) The Mises stress of the fiber network and a partially enlarged detail of it at the initial state. All fibers were merged into a single part. The end face at z = 0 was fully constrained, while a reference point (RP) was created at the centroid of the opposite end face (z = z_length) with prescribed z-direction displacement applied. (**c**) The Mises stress of the fiber network at the end state. A partially enlarged detail shows that mesh elements underwent large deformations.

Given the limited sample size for multivariate linear regression analysis and the influence of confounding factors (e.g., fiber diameter distribution and bonding), finite element modeling was subsequently adopted to simulate the mechanical response of TPU non-woven fabrics under tensile loading. As shown in Figure 3a, a 3D model of fibers was generated within a defined rectangular space by Python code and then meshed with hexahedral elements. Figure 3b shows the Mises stress distribution of the fiber network before and after stretch. During uniaxial stretching, TPU non-woven fibers exhibit a localized necking phenomenon, resulting in stress concentration effects.

**Figure 4:**
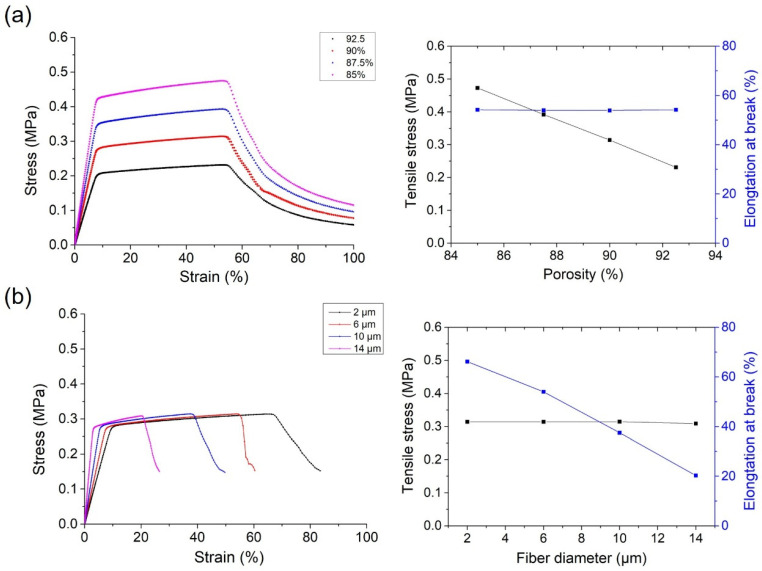
Tensile behaviors of melt-blown TPU fabrics simulated by FEM. (**a**) Simulated tensile curves and tensile properties of non-woven fabrics with porosity changed from 85% to 92.5%, while fiber diameter was 6 μm. (**b**) Simulated tensile curves and tensile properties of non-woven fabrics with fiber diameter changed from 2 μm to 14 μm, while porosity was 90%. Here, the tensile stress is defined as the maximum stress during tensile, and elongation at break is defined as the strain corresponding to it.

To analyze the influence of the structure of the fiber network on the mechanical behavior of non-woven fabrics, fiber diameter or porosity was changed, while the other factors were kept the same. As shown in Figure 4a, when porosity was increased from 85% to 92.5%, the tensile stress declined from 0.47 MPa to 0.21 MPa, while elongation at break was almost the same at 54%. In Figure 4b, when the fiber diameter was increased from 2 μm to 14 μm, elongation at break declined from 66.2% to 20.3%, while tensile stress was almost the same, around 0.31 MPa.

## 4. Discussion

### 4.1. Characterization and Melt-Blowing of TPU Material

The appropriate selection of TPU raw material is the cornerstone of the successful fabrication of non-woven fabrics. FTIR analysis revealed a microphase-separated structure in the TPU raw material (Figure 1a), with a calculated degree of microphase separation of 35%. This structural feature is critical, as it directly influences the elasticity and mechanical performance of the material, which are essential for applications requiring flexibility and durability, such as wound dressings. Owing to the microphase-separated structure, the TPU material showed high elasticity, with elongation at break surpassing 700% (Figure 1b).

The melt-blowing process itself presents unique challenges due to TPU’s temperature-sensitive rheology and complex chemical structure. Unlike polypropylene, which dominates commercial melt-blown non-wovens, TPU requires precise control of parameters such as melt temperature, air pressure, and die-to-collector distance to achieve optimal fiber attenuation and deposition. The apparatus for melt-blowing is illustrated in Figure 2a,b. The high-velocity hot air stretches the extruded polymer into ultrafine fibers (1–10 μm), while the semi-molten state of TPU facilitates fiber entanglement and bonding during collection. These factors collectively contribute to the formation of a three-dimensional fibrous network with tailored porosity and mechanical properties, making TPU non-wovens ideal for advanced medical and industrial applications. This research mainly focuses on fibrous network architecture, such as fiber diameter and porosity. However, future work could extend the analysis to the aggregation structure of fiber, combining techniques such as XRD (X-ray Diffraction) to investigate molecular-level organization within fibers.

### 4.2. Relationship Between Microstructure and Macroscopic Properties of Melt-Blown TPU Fabrics

Due to the inherent heat sensitivity of the TPU, fabrication of TPU fabrics by melt-blowing is much more difficult than traditional polyolefin fabrics. Some studies have discussed how process parameters influence TPU fabric properties [27,39], primarily focusing on the influence of processing parameters on the performance of non-woven fabrics. However, processing parameters exert their effects by modulating fibrous network architecture, which subsequently determines the macroscopic properties of the non-woven materials.

Therefore, our research systematically investigated the direct relationship between fiber architecture and the overall performance of non-woven fabrics. By controlling process parameters, mainly die-to-collector distance and hot air frequency, TPU non-woven fabrics with fiber diameters ranging from 7.4 μm to 9.5 μm and porosity ranging from 87.1% to 90.1% were prepared (Table 1). Despite the difference in fiber diameter and porosity, the grammage was kept almost the same, around 75%.

Multiple linear regression was then employed to quantitatively analyze the relationships between fiber structural parameters (diameter, porosity, and grammage) and the mechanical properties of TPU non-woven fabrics, as this statistical approach enables simultaneous evaluation of multiple variables while accounting for their potential interactions through standardized coefficients (*β*). The analysis results in Table 2 and Table 3 reveal that grammage is the most important factor for tensile behavior except for longitudinal elongation at break. This makes sense because grammage is directly associated with the thickness of non-woven fabrics. The different effects of structural factors on transverse and longitudinal mechanical properties are primarily attributed to the fiber orientation anisotropy and strain-induced molecular alignment during melt-blown processing, which create distinct load-bearing mechanisms along different directions.

However, several critical limitations about MLR must be considered when interpreting these results [40,41]: the small sample size significantly constrained statistical power and resulted in large standard errors, making conventional significance testing unreliable; the inherent linear assumptions of MLR may oversimplify the potentially nonlinear relationships in fiber-network mechanics; and unaccounted confounding factors like fiber orientation distribution could bias the coefficients.

### 4.3. Establishment of Finite Element Modeling with 3D Fiber Network to Analyze Mechanical Behaviors of Non-Woven Fabrics

Recent advances in computational and analytical models, such as artificial neural networks, numerical analysis, FEM, and so on [42,43,44,45,46,47,48], have deepened the understanding of microstructure–property relationships in nano- or microfibrous mats. Among these, FEM of non-woven fabrics with 2D finite element models of fiber networks has clear advantages in computational efficiency and modeling simplicity, particularly for analyzing in-plane mechanical properties of fibrous mats.

However, this approach inevitably neglects important thickness-direction effects and complex three-dimensional interactions within the fiber network. In contrast, a 3D model provides more accurate simulations of spatial fiber behaviors, such as entanglement and local buckling, but its implementation faces significant challenges, including substantially higher computational costs and the need to address complex contact mechanics and nonlinear problems, especially for materials with low elastic modulus and ultra-high elongation at break, like TPU. To address this, a 3D model generated by Python code was used for FEM (Figure 3a). Hexahedral meshes offer superior computational efficiency with fewer elements while accurately capturing transverse stress. A swept mesh was generated by extruding a quadrilateral-dominant surface mesh from the fiber’s cross-section along its axis, discretizing both the circumference and length into multiple elements. Results of this model provide more visual details for the distribution of stress on the fiber network (Figure 3c). Data show that tensile stress and elongation at break of non-woven fabrics are closely related to porosity and fiber diameter, respectively (Figure 4).

A quantitative discrepancy, which is reasonable and expected given the simplifications inherent in computational modeling, is observed between the FEM predictions and the experimental results. For example, the FEM result of tensile strength and elongation at break of samples with a fiber diameter of 10 μm and porosity of 90% is about 0.31 MPa and 38%, while the experimental results of Sample 1 with a similar structure are about 1.41 MPa and 430%. The quantitative differences between the simulated and experimental tensile properties arise from necessary simplifications in the FEM. The model employs an idealized network with uniform fiber diameter and idealized elastoplastic material behavior and omits inter-fiber bonding—factors that are present in the real fabric and contribute to its higher strength and elongation [49,50]. Future model iterations incorporating diameter distributions, cohesive bonding, and hyperelastic material laws would enhance predictive accuracy. Nonetheless, the current model successfully captures the fundamental directional trends imposed by porosity and diameter, providing mechanistic validation for the experimental structure–property relationships.

## 5. Conclusions

This study systematically investigated the influence of fibrous network architecture on the mechanical and physical properties of melt-blown TPU non-woven fabrics. We first demonstrated the critical role of TPU’s microphase-separated structure in achieving high elasticity and mechanical durability, which are essential for applications such as wound dressings. Through precise control of melt-blowing parameters, mainly by air speed and die-to-collector distance, fabrics with varying fiber diameters and porosities were fabricated, enabling a detailed analysis of structure–property relationships. Our findings reveal that grammage significantly impacts tensile strength, while fiber diameter and porosity play different roles in transverse and longitudinal tensile behavior. Notably, finite element modeling of 3D fiber networks further validated these relationships, demonstrating that increased porosity reduces tensile stress, whereas larger fiber diameters decrease elongation at break. By combining experimental characterization with computational modeling, this study offers practical guidelines for tailoring the hierarchical structure of TPU non-wovens to meet specific performance requirements in medical and industrial applications.

## Figures and Tables

**Table 1:** The structural properties (grammage, fiber diameter, and porosity) and corresponding mechanical properties of melt-blown non-woven fabrics. S1 to S5 represent different melt-blown samples.

c	Unit	S1	S2	S3	S4	S5
Grammage	g/m^2^	74.6 ± 0.7	75.1 ± 1.2	75.3 ± 1.5	74.7 ± 0.6	74.3 ± 1.0
Fiber diameter	μm	9.52 ± 0.82	8.46 ± 0.55	8.32 ± 0.43	7.47 ± 0.26	9.05 ± 0.61
Porosity	%	90.1 ± 0.4	88.6 ± 0.7	87.1 ± 0.2	87.8 ± 0.3	87.6 ± 0.5
Tensile force (transverse)	N/5 cm	21.2 ± 1.5	19.8 ± 2.1	21.9 ± 1.2	20.1 ± 1.8	16.2 ± 0.9
Tensile force (longitudinal)	N/5 cm	23.9 ± 1.9	26.6 ± 2.6	25.9 ± 1.7	20.6 ± 2.2	23.3 ± 1.7
Elongation at break (transverse)	%	430 ± 62	438 ± 37	401 ± 50	369 ± 71	346 ± 66
Elongation at break (longitudinal)	%	438 ± 59	392 ± 65	375 ± 77	371 ± 41	401 ± 53
Air permeability	mm/s	290 ± 6	225 ± 5	113 ± 2	126 ± 2	119 ± 6

**Table 2:** The influence of fibrous network architecture on the transverse tensile behavior of melt-blown fabrics analyzed by multiple linear regression. Here, coefficients refer to standardized regression coefficients.

Parameters	Tensile Force		Elongation at Break	
	Coefficient (*β*)	Standard Error	Coefficient (*β*)	Standard Error
Fiber diameter (μm)	−0.112	2.0	0.152	3.0
Porosity (%)	0.424	1.7	0.756	2.5
Grammage (g/m^2^)	0.764	3.5	0.774	5.0

**Table 3:** The influence of fibrous network architecture on the longitudinal tensile behavior of melt-blown fabrics analyzed by multiple linear regression. Here, coefficients refer to standardized regression coefficients.

Parameters	Tensile Force		Elongation at Break	
	Coefficient (*β*)	Standard Error	Coefficient (*β*)	Standard Error
Fiber diameter (μm)	0.758	1.5	0.600	5.1
Porosity (%)	−0.133	1.3	0.458	4.4
Grammage (g/m^2^)	0.899	2.6	−0.152	8.7

## Data Availability

The original contributions presented in this study are included in the article/Supplementary Material. Further inquiries can be directed to the corresponding authors.

## Supplementary Materials

The following supporting information can be downloaded at https://www.mdpi.com/article/10.3390/polym18070857/s1, Figure S1. Result of fiber orientation. (a) Typical SEM images of fiber with low magnification. (b) Statistic result of degree of fiber orientation; Figure S2. FTIR of melt-blown non-woven samples. (a–e) Sample 1 to 5.
