# Supplementary material for: Influence of the Fibrous Network Architecture on the Mechanical Properties of Melt-Blown Non-Woven Thermoplastic Polyurethane Fabrics
Source: Polymers (Basel). 2026 Mar 31;18(7):857. doi: 10.3390/polym18070857 (PMC13074388; doi:10.3390/polym18070857)
Supplement: Supplementary file 1 [file polymers-18-00857-s001.zip › polymers-4095237-supplementary.pdf]

Influence of the Fibrous Network Architecture on the Mechanical Properties of Melt-Blown Non-Woven Thermoplastic Polyurethane Fabrics

Qunsong Wang[1,2], Ming Lu[2], Rimin Zhou[2], Mingkun Li[2], Chao Ding[2], Yun Liang[1*]*

1 National engineering research center of papermaking and pollution control, South China University of Technology, Guangzhou 510641, China.

2 Kingfa Sci.&Tech. Co.,Ltd, Guangzhou 510663, China.

* Corresponding author. Email: liangyun@scut.edu.cn (Y Liang), dingchao@kingfa.com.cn (C Ding)

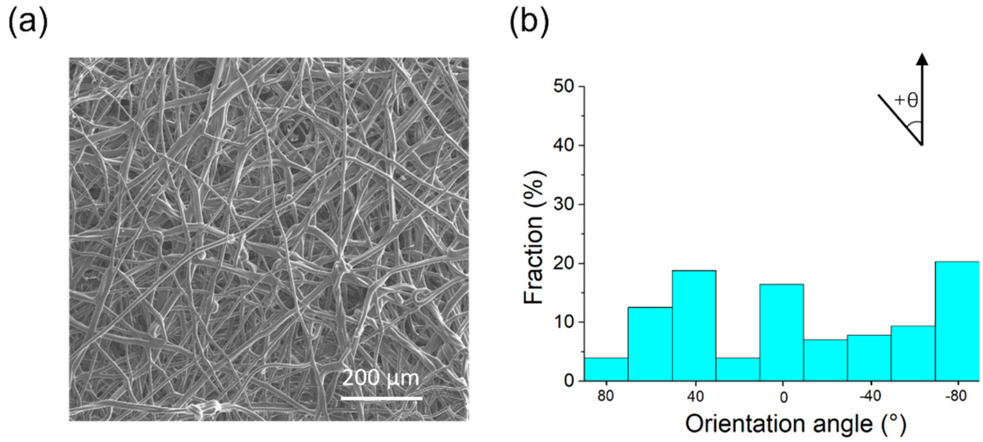

Figure. S1 Result of fiber orientation. (a) Typical SEM images of fiber with low magnification. **(b)** Statistic result of degree of fiber orientation.

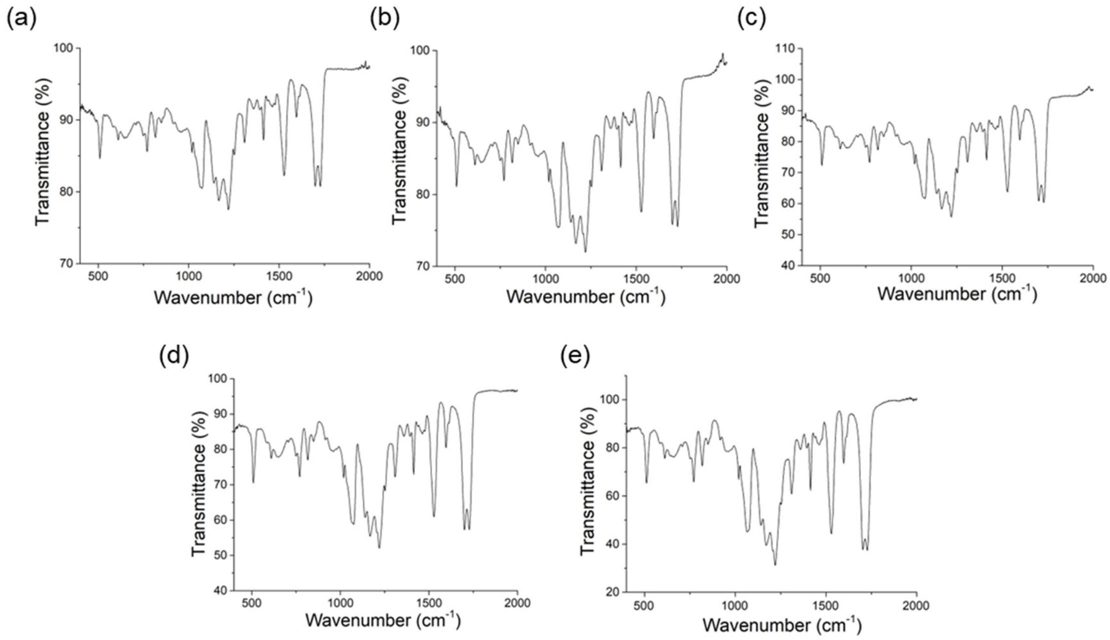

Figure S2. FTIR of melt-blown non-woven samples. (a-e) Sample 1 to 5**.**
